# Functional brain imaging and central control of the bladder in health and disease

**DOI:** 10.3389/fphys.2022.914963

**Published:** 2022-08-12

**Authors:** Dongqing Pang, Yi Gao, Limin Liao

**Affiliations:** ^1^ China Rehabilitation Research Center, School of Rehabilitation, Capital Medical University, Beijing, China; ^2^ Department of Urology, China Rehabilitation Research Center, Beijing, China; ^3^ Department of Urology, Capital Medical University, Beijing, China

**Keywords:** bladder, urination, functional magnetic resonance imaging, near-infrared spectroscopy, brain mapping

## Abstract

Central control of the bladder is a complex process. With the development of functional imaging technology and analysis methods, research on brain-bladder control has become more in-depth. Here, we review previous functional imaging studies and combine our latest findings to discuss brain regions related to bladder control, interactions between these regions, and brain networks, as well as changes in brain function in diseases such as urgency urinary incontinence, idiopathic overactive bladder, interstitial cystitis/bladder pain syndrome, urologic chronic pain syndrome, neurogenic overactive bladder, and nocturnal enuresis. Implicated brain regions include the pons, periaqueductal grey, thalamus, insula, prefrontal cortex, cingulate cortex, supplementary motor area, cerebellum, hypothalamus, basal ganglia, amygdala, and hippocampus. Because the brain is a complex information transmission and processing system, these regions do not work in isolation but through functional connections to form a number of subnetworks to achieve bladder control. In summarizing previous studies, we found changes in the brain functional connectivity networks related to bladder control in healthy subjects and patients involving the attentional network, central executive network or frontoparietal network, salience network, interoceptive network, default mode network, sensorimotor network, visual network, basal ganglia network, subcortical network, cerebella, and brainstem. We extend the working model proposed by Griffiths et al. from the brain network level, providing insights for current and future bladder-control research.

## Introduction

Understanding brain-bladder control mechanisms in healthy adults is essential to identifying the central abnormalities in patients with lower urinary tract dysfunction (LUTD). The lower urinary tract has two main functions: urine storage and micturition. Switching between the two phases depends on current bladder capacity, external environment, and volitional control. The supraspinal, spinal, autonomic, and somatic nerve pathways work together to control the balance between these two functions. In 2008, Fowler et al. (2008) proposed the neural circuits that control continence and micturition. Specifically, there are two reflexes: 1) Urine storage reflexes, which occur mainly in the spinal cord and are important in the urine-storage phase. During bladder filling, bladder distention generates low levels of bladder-afferent signals that stimulate the hypogastric and pudendal nerves to contract the bladder outlet, inhibit the contraction of the detrusor, and contract the external urethral sphincter, respectively; and 2) Voiding reflexes, in which the intense afferent signal from the bladder during urination activates the pontine micturition center (PMC), which increases the efferent activity of the parasympathetic nerve and contracts the detrusor muscle, while inhibiting efferent activity of the sympathetic and pudendal nerves to the urethral outlet. Upstream afferent signals from the spinal cord may reach the PMC through the periaqueductal grey (PAG).

People can urinate voluntarily when it is convenient or delay urination when it is inconvenient, which may rely on brain circuits above the PAG. (Fowler et al., 2008). Because previous articles have reviewed PAG and pons in detail, (de Groat et al., 2015), we focused on brain regions above PAG, especially the interaction between brain regions and networks.

### Brain regions related to bladder control

The idea that the brain may be involved in bladder control was supported by clinical experience. As early as 1964, Andrew and Nathan found that prefrontal cortex (PFC) lesions caused by issues such as tumors, aneurysms, wounds, and leukotomy, which may lead to an impaired ability to inhibit the voiding reflex, resulting in urinary urgency and incontinence, suggesting that the PFC is crucial for bladder control. (Andrew and Nathan, 1964). In 1996, Sakakibara (Sakakibara et al., 1996) analyzed the cranial computed tomography (CT) or magnetic resonance imaging (MRI) of 72 patients with acute hemispheric stroke and their urodynamic examination results, finding that patients with frontal lobe lesions showed detrusor hyperreflexia and uninhibited sphincter relaxation, leading to lower urinary tract symptoms (LUTS) such as frequency and urgency urinary incontinence (UUI) in stroke patients.

In the past few decades, with the rapid development of functional brain imaging technology, single-photon emission CT (SPECT) and positron emission tomography (PET) technologies have been gradually applied in brain-bladder research. These methods can indirectly reflect local brain neural activity by measuring regional cerebral blood flow (rCBF) changes in brain regions. (Kitta et al., 2015). Fukuyama et al. (1996) first used SPECT in 1996 to search for brain regions with activity potentially correlated to urination control in healthy subjects. In 1997, Blok et al. (1997) first performed a 15O-H2O PET study of healthy men during storage, micturition, and post-micturition. They conducted the same study in 1998 in healthy women, finding the same changes (i.e., significantly increased blood flow in the inferior frontal gyrus, pons, and PAG). They suggested that the micturition may be associated with these brain regions. (Blok et al., 1998). In 2000, Nour et al. (2000) first applied the experimental paradigm of bladder filling by means of bladder perfusion, in which the bladder was filled by perfusing normal saline into the intravesical catheter. They were the first to try a PET scan with simultaneous detrusor pressure monitoring. PET scans were performed during an empty bladder, full bladder (normal desire to void), and micturition.

Subsequently, two other brain functional imaging techniques, namely functional magnetic resonance imaging (fMRI) and functional near-infrared spectroscopy (rs-fNIRS), have been used to study the hypothetical central mechanism of bladder control. fMRI is based on the quantification of paramagnetism of deoxy-Hb and has the advantages of high temporal and spatial resolution. (Kitta et al., 2015). The physiological processes underlying BOLD fMRI imaging are neurovascular coupling mechanisms. (Stackhouse and Mishra, 2021). Specifically, increased synaptic activity leads to the dilation of local arterioles and an increase in cerebral blood flow (CBF) to match enhanced metabolic demands and maintain normal brain function. (Munoz et al., 2015). fNIRS is based on the absorption of near-infrared light by oxyhemoglobin (oxy-Hb) and deoxy-Hb and has the advantages of portability and high temporal resolution. (Jobsis, 1977).

Previous studies have shown that activation of brain regions shows increased rCBF and glucose utilization but little increase in oxygen utilization, resulting in an increase in the amount of oxygen available in the active regions (i.e., oxy-Hb), which explains the blood oxygen level-dependent (BOLD) signal of fMRI and fNIRS, while deactivation shows an opposite pattern of change. (Raichle et al., 2001). Because both excitatory and inhibitory neural activity increase local glucose utilization in animal models, (Ackermann et al., 1984; Batini et al., 1984), deactivation is unlikely to result from increased activity of locally inhibitory neurons. Deactivation, in contrast, may indicate a reduction in nerve cell activity to a level below baseline.

In 2015, Griffiths et al. (2015a) (de Groat et al., 2015) reviewed previous brain functional imaging studies and proposed a working model related to bladder control. Specifically, circuit one includes the thalamus, insula, lateral PFC (LPFC), medial PFC (MPFC), and PAG. Griffiths et al. (2015a) suggested a possible mechanism for maintaining continence in healthy subjects: the sensation of bladder dilation is uploaded to the insula *via* the thalamus, which activates LPFC and in turn reduces MPFC activity through an inhibitory connection, reducing MPFC input to PAG, stabilizing PAG and inhibiting the voiding reflex and, finally, maintaining continence.

Circuit 2 includes the dorsal anterior cingulate cortex (dACC) and supplementary motor area (SMA). The team suggested that this circuit is a backup used by people with UUI or overactive bladder (OAB) rather than healthy subjects. That is, if there is a threat of urinary incontinence, dACC and SMA seem to respond by producing a sense of urgency and urinary sphincter contraction, enhancing the ability to delay urination. Circuit 3 includes the PAG and parts of the inferior or middle temporal (parahippocampal) cortex, whose role may be to provide unconscious monitoring during low bladder volume/low bladder sensation in healthy subjects.

### Urine storage phase

Most previous functional brain imaging studies focused on changes in brain activity during the urine storage phase. These studies achieved a full bladder/desire to void by having participants drink water (Fukuyama et al., 1996; Blok et al., 1997; Gao et al., 2015; Pang et al., 2020; Pang and Liao, 2021) and performing repeated perfusion/withdrawal of saline through the urinary catheter, (Griffiths et al., 2005; Matsumoto et al., 2011; Pang et al., 2021), or repeated spontaneous contraction of pelvic floor muscles (Kuhtz-Buschbeck et al., 2005; Zhang et al., 2005; Seseke et al., 2006) to activate relevant brain regions. Our recent fMRI study (Pang et al., 2021) found that regional homogeneity (ReHo) values in the thalamus, insula, medial frontal gyrus, and bilateral superior frontal gyrus of healthy adults changed significantly when the bladder was full rather than empty, which support the theory of this model. Sakakibara et al. (1996) found that LUTS might be associated with thalamus impairment in stroke patients after the analysis of the lesion site by CT and MRI. Previous studies have shown that bladder filling, leading to a desire to void, may significantly activate the insula in healthy subjects, and activation increases with the degree of bladder filling and desire to void. (Kuhtz-Buschbeck et al., 2005). Moreover, in OAB patients, insula activation was exaggerated during bladder filling, (Griffiths et al., 2005; Tadic et al., 2010), so the insula was considered related to bladder distention and the desire to void. The interoceptive visceral sensation (bladder distention) ^27^is uploaded through the spinal cord and then relayed in the thalamus, targeting the insula, the interoceptive afferent cortex. (Blok et al., 1997; Craig, 2003).

The PFC, located in brain regions in front of the primary and premotor cortex, including the superior frontal gyrus, middle frontal gyrus, and inferior frontal gyrus, was shown to be related to bladder control in previous neuroimaging studies. (Nour et al., 2000; Griffiths et al., 2009). Previous studies showed MPFC deactivation (Griffiths et al., 2007; Griffiths et al., 2009) and LPFC activation when the bladder was full during urine storage. (Kuhtz-Buschbeck et al., 2005; Yin et al., 2008; Sakakibara et al., 2010; Matsumoto et al., 2011). The deactivation of MPFC is thought to be related to maintaining urine storage. (Blok et al., 1997; Blok et al., 1998; Griffiths et al., 2007; Griffiths et al., 2009). Andrew and Nathan generalize their idea of a possible center (i.e., the medial, periventricular, mid-frontal region) that might control the function of urination after reviewing a case series that included an abundance of leukotomy patients. They found that permanent urination problems may be associated with large lesions and severe lesions. (Andrew and Nathan, 1964). Deactivation of the MPFC may indicate reduced neural activity of the MPFC to below baseline. (Raichle et al., 2001). MPFC is an important part of the default mode network (DMN), which is characterized by activation of brain areas within the network in the resting state (i.e., eyes closed or simple visual fixation) and deactivation in the presence of external stimuli. (Raichle et al., 2001; Fox and Raichle, 2007). Deactivation of a brain region within DMN indicates that resting activity is suspended while the brain uses its resources to process events that require conscious attention.

But until now, it has not been clear whether the deactivation of MPFC during urine storage phase is the cause of urinary incontinence or the mechanism by which the urination reflex is suppressed and continence is maintained. Griffiths believes the latter is the brain response of people trying to avoid improper bladder contractions. (Griffiths, 2015b). The fear of public incontinence during urinary urgency can cause tension related to social etiquette, with increased heart rate. Previous studies have shown that this increased heart rate caused by social evaluative threat may be mediated by activation of rostral dorsal ACC and deactivation of ventral mPFC. (Wager et al., 2009). Griffiths suggested that the bladder control during urinary urgency may be achieved by using a dorsal ACC-based sympathetic mechanism and MPFC-based parasympathetic mechanism. (Griffiths, 2015a). PFC is involved in human higher cognitive function, and the brain region responsible for executive function is mainly the bilateral dorsolateral prefrontal cortex (DLPFC), including BA9, 10, and 46. Executive functions include organizing input from different sensory modes, maintaining attention, monitoring information in working memory, and coordinating goal-directed behavior. (Teffer and Semendeferi, 2012). Duffau and Capelle (2005) reported two cases of patients with long-term UUI after glioma resection, considering that DLPFC lesions confirmed by structural MRI may result in an inability to delay voiding. Previous studies have shown that the stronger the desire to void, the stronger the activation of bilateral DLPFC, suggesting that DLPFC may be related to the monitoring of interoceptive stimulation and the perception of bladder sensation. (Kuhtz-Buschbeck et al., 2005; Matsumoto et al., 2011).

### Micturition phase

Compared with the urine storage stage, relatively few studies have investigated changes in brain activity during micturition, including one SPECT, (Fukuyama et al., 1996), three PET, (Blok et al., 1997; Blok et al., 1998; Nour et al., 2000), and two fMRI studies. (Krhut et al., 2012; Shy et al., 2014). The task paradigm of repeated micturition was first used in 2012. (Krhut et al., 2012). By summarizing information on the brain regions involved in these studies for the first time (Table 1), we found that the frequency of activation of brain regions during micturition was five times for the LPFC/inferior frontal gyrus and pons; three times for PAG, hypothalamus, basal ganglia, and anterior cingulate cortex (ACC); twice for the postcentral gyrus, thalamus, insula, superior frontal gyrus, cerebellar, and SMA. This suggests that these brain regions play an important role in the micturition phase.

**TABLE 1:** Brain areas activated during micturition.

Brain areas activated during micturition	Authors	Subjects	Functional imaging technique
Lateral PFC/inferior frontal gyrus	Fukuyama et al. (1996)	Healthy men	SPECT
	Blok et al. (1997)	Healthy men	PET
	Blok et al. (1998)	Healthy women	PET
	Nour et al. (2000)	Healthy men	PET
	Krhut et al. (2012)	Healthy women	fMRI
Pons/PMC	Fukuyama et al. (1996)	Healthy men	SPECT
	Blok et al. (1997)	Healthy men	PET
	Blok et al. (1998)	Healthy women	PET
	Nour et al. (2000)	Healthy men	PET
	Shy et al. (2014)	Healthy women	fMRI
PAG	Blok et al. (1997)	Healthy men	PET
	Blok et al. (1998)	Healthy women	PET
	Nour et al. (2000)	Healthy men	PET
Hypothalamus	Blok et al. (1997)	Healthy men	PET
	Blok et al. (1998)	Healthy women	PET
	Nour et al. (2000)	Healthy men	PET
Basal ganglia	Blok et al. (1997) (striatum)	Healthy men	PET
	Nour et al. (2000) (globus pallidus)	Healthy men	PET
	Shy et al. (2014) (caudate nucleus and lentiform nucleus)	Healthy women	fMRI
ACC	Blok et al. (1997)	Healthy men	PET
	Krhut et al. (2012)	Healthy women	fMRI
	Shy et al. (2014)	Healthy women	fMRI
Postcentral gyrus	Nour et al. (2000)	Healthy men	PET
	Shy et al. (2014)	Healthy women	fMRI
Thalamus	Nour et al. (2000)	Healthy men	PET
	Shy et al. (2014)	Healthy women	fMRI
Insula	Nour et al. (2000)	Healthy men	PET
	Shy et al. (2014)	Healthy women	fMRI
Superior frontal gyrus/mPFC	Nour et al. (2000)	Healthy men	PET
	Shy et al. (2014)	Healthy women	fMRI
Cerebellar	Nour et al. (2000)	Healthy men	PET
	Shy et al. (2014)	Healthy women	fMRI
SMA	Fukuyama et al. (1996)	Healthy men	SPECT
	Shy et al. (2014)	Healthy women	fMRI

### Interactions between brain regions and networks

In addition to studying the activation/deactivation of a specific brain region associated with bladder control, some studies over the past 20 years have focused on the communication, collaboration, separation, and integration of brain regions. Functional connectivity (FC) refers to the display of coherent neural activity in anatomically isolated brain regions. The FC of different paired brain regions together constitute brain functional networks. (Ketai et al., 2016; Zuo et al., 2019b). By summarizing these studies, we found changes in the brain FC and brain network related to bladder control in healthy subjects and patients with LUTD, including the following brain networks (Table 2): Attentional network (AN), central executive network (CEN) or frontoparietal network (FPN), salience network (SN) or interoceptive network (IN), default mode network (DMN), sensorimotor network (SMN), visual network (VN), basal ganglia network (BGN), subcortical network, cerebella, and the brainstem.

**TABLE 2:** Changes in the brain FC and networks related to bladder control in healthy subjects and patients with LUTD.

Network	Description anatomic areas and function	Activation or FC changes within the network	Authors	Subjects
Attentional network, AN	**Ventral** AN includes the temporoparietal junction (TPJ) and ventral frontal/prefrontal cortex. **Ventral** AN response to unexpected events (bottom-up attention) Vossel et al. (2014)	Bladder distention increased the activation of brain regions within the ventral AN (bilateral TPJ). (task-fMRI)	Jarrahi et al. (2015a)	Healthy women
		The ventral AN (left supramarginal gyrus) were significantly activated in healthy women with a full bladder compared with an empty bladder. (task-fMRI)	Nardos et al. (2014)	Healthy women
		Compared to the baseline before treatment, combined groups showed decreased activation of the left TPJ. (task-fMRI)	Ketai et al. (2021)	UUI women; hypnotherapy VS. pharmacotherapy
		Compared to HC, UUI group showed greater activation within the v**entral** AN (i.e., VLPFC, bilateral middle superior temporal and supramarginal gyrus). (task-fMRI)	Ketai et al. (2016)	UUI women VS. HC
	**Dorsal** AN includes the bilateral frontal eye field and the intraparietal sulcus	Compared to pharmacotherapy, hypnotherapy participants manifested increased functional connectivity (FC) within **dorsal** AN. (rs-fMRI)	Ketai et al. (2016)	UUI women; hypnotherapy VS. pharmacotherapy
	**Dorsal** AN response to goal-directed (top-down attention) processing. Vossel et al. (2014)			
		FC within the **dorsal** DAN (i.e., precentral gyrus) was significantly decreased in OAB group compared with HC. (rs-fMRI)	Zuo et al. (2019a)	OAB patients VS. HC
Central executive network (CEN) or frontoparietal network (FPN)	CEN consists of the DLPFC and the lateral posterior parietal cortex. Chan et al. (2016)	Bladder distention increased the activation of brain regions within the CEN (DLPFC and posterior parietal cortices). (task-fMRI)	Jarrahi et al. (2015a)	Healthy women
	CEN is responsible for active maintenance and manipulation of information in working memory, as well as judgment and decision making under goal-directed behavior. Chan et al. (2016)	Compared to HC, OAB group showed increased FC strength in middle frontal gyrus, which is a part of DLPFC. (rs-fMRI)	Zuo et al. (2019b)	OAB patients VS. HC
		FC within the LFPN (i.e., superior frontal gyrus) was significantly decreased in OAB group compared with HC. (rs-fMRI)	Zuo et al. (2019a)	OAB patients VS. healthy controls
		UUI patients had significantly abnormal activation within CEN (i.e., inferior and superior frontal gyrus) compared with HC. (task-fMRI)	Nardos et al. (2016)	UUI women VS. HC
Salience network (SN) or interoceptive network (IN)	SN includes the ACC and the anterior insula. Seeley et al. (2007)	Compared to empty bladder, we found increased ReHo in the brain region (i.e., left insula and bilateral ACC) within SN with a full bladder. (rs-fMRI)	Pang et al. (2021)	Healthy subjects; full bladder VS. empty bladder
	SN is responsible for locating and detecting associated stimuli. Seeley et al. (2007)			
		Bladder distention increased the activation of brain regions within the SN (anterior insula and ACC). (task-fMRI)	Jarrahi et al. (2015a)	Healthy women
		The SN (i.e., left ACC) were significantly activated in healthy women with a full bladder compared with an empty bladder. (task-fMRI)	Nardos et al. (2014)	Healthy women
		Compared to HC, UUI group showed greater activation within the Interoceptive network (i.e., left island and ACC). (task-fMRI)	Ketai et al. (2016)	UUI women VS. HC
Default mode network, DMN	DMN includes the posterior cingulate cortex (PCC), the medial prefrontal cortex (MPFC), the precuneus, the medial temporal lobe, and the **AG**. Fox and Raichle (2007)	Compared to empty bladder, we found increased ReHo in the brain region (i.e., left temporal gyrus and left **AG**) within DMN with a full bladder. (rs-fMRI)	Pang et al. (2021)	Healthy subjects; full bladder VS. empty bladder
	DMN is involved in social or self-referential processing, stimulus-independent thought, manipulation of episodic memories, and semantic knowledge. Chan et al. (2016)	Compared to empty bladder, significantly increased FC within DMN (i.e., superior frontal gyrus, PCG, and AG) when the desire to void was strong. (rs-fMRI)	Pang et al. (2020)	Healthy subjects; strong desire to void VS. empty bladder
		Bladder distention increased the activation of brain regions within the DMN (MPFC, the precuneus/PCC, bilateral parietal lobules, and the inferior temporal gyri). (task-fMRI)	Jarrahi et al. (2015a)	Healthy women
		Compared to HC, OAB group showed decreased FC strength in hubs of the DMN (eg the PCG and the MPFC). (rs-fMRI)	Zuo et al. (2019b)	OAB patients VS. HC
		UUI patients had significantly abnormal activation within DMN (i.e., inferior parietal lobe) compared with HC. (task-fMRI)	Nardos et al. (2016)	UUI women VS. HC
		Compared to HC, UUI group showed greater activation within the posterior DMN (i.e., PCC and precuneus). (task-fMRI)	Ketai et al. (2016)	UUI women VS. HC
		Compared to HC, urologic chronic pain syndrome (UCPPS) group showed that the FC of DMN was significantly reduced to PCC and left precuneus. (rs-fMRI)	Martucci et al. (2015)	women with UCPPS VS. HC
Sensorimotor network, SMN	SMN includes the somatosensory area, the primary motor cortex, the secondary motor cortex, the SMA, and the premotor cortex. Chan et al. (2016)	Compared to empty bladder, strong desire to void group showed an increased nodal efficiency in the SMN (i.e., bilateral postcentral gyrus). (rs-fMRI)	Pang et al. (2020)	Healthy subjects; strong desire to void VS. empty bladder
	SMN has pre-mediated functions that coordinate the functions of multiple brain regions in preparation for motor responses to sensory input. Zuo et al. (2019a)	FC within the SMN (i.e., paracentral lobule) was significantly decreased in OAB group compared with HC. (rs-fMRI)	Zuo et al. (2019a)	OAB women VS. healthy controls
		UUI patients had significantly abnormal activation within SMN (i.e., precentral and postcentral gyrus) compared with HC. (task-fMRI)	Nardos et al. (2016)	UUI women VS. HC
Visual network, VN	VN located in the visual cortex and is divided into dorsal VN and ventral VN.	Compared to empty bladder, strong desire to void group showed an increased nodal efficiency in the VN (i.e., superior occipital gyrus, bilateral middle occipital gyrus, and cuneus). (rs-fMRI)	Pang et al. (2020)	Healthy subjects; strong desire to void VS. empty bladder
	The dorsal VN processes information about the position of objects and adjusts visual controls for skilled movements. Migliaccio et al. (2016)			
		FC within the dorsal VN (i.e., left cuneus) was significantly decreased in OAB group compared with HC. (rs-fMRI)	Zuo et al. (2019a)	OAB patients VS. healthy controls with empty bladder
Basal ganglia network, BGN	BGN includes the striatum, consisting of caudate nucleus and lenticular nucleus (including putamen and globus pallidus), claustrum, amygdala, red nucleus, substantia nigra, and subthalamic nucleus. (Smitha et al., 2017)	Compared to empty bladder, strong desire to void group showed an increased nodal efficiency in the BGN (i.e., caudate nucleus). (rs-fMRI)	Pang et al. (2020)	Healthy subjects; strong desire to void VS. empty bladder
	BGN is responsible for the process of motor areas control, emotion, cognition, etc. They engage in goal-directed behavior that requires movement. (Smitha et al., 2017)	Bladder distention increased the activation of brain regions within the BGN (bilateral striatum and amygdala) and thalamus. (task-fMRI)	Jarrahi et al. (2015a)	Healthy women
		Patients with UUI who responded to pelvic floor muscle therapy (PFMT) had significant differences in FC of BG (caudate nucleus and putamen), thalamus, and dACC compared with before treatment. (rs-fMRI)	Clarkson et al. (2018)	UUI women who responded VS. non-responded to PFMT

### Healthy subjects

Some studies on healthy subjects could serve as a basis for explaining brain abnormalities in patients with LUTD. We found that healthy subjects showed increased ReHo in the brain region within DMN (i.e., left superior temporal gyrus and left angular gyrus [AG]) and SN with a full bladder, compared with an empty bladder. (Pang et al., 2021). Moreover, we found significantly increased FC within DMN (i.e., superior frontal gyrus, posterior cingulate cortex [PCC], and AG), and increased nodal efficiency (enodal) in the BGN (i.e., caudate nucleus), DMN (i.e., PCC), SMN (i.e., bilateral postcentral gyrus), and VN (i.e., superior occipital gyrus, bilateral middle occipital gyrus, and cuneus) in healthy subjects when the desire to void was strong versus an empty bladder. (Pang et al., 2020). We suggested that that SN provides bladder sensation and that DMN may provide self-reference, self-refection, and decision-making about whether to void after assessment of the external environment. Moreover, the bladder-control process may be coordinated by multiple subnetworks (e.g., BG, SMN, VN). (Pang et al., 2020; Pang et al., 2021). Nardos et al. (2014) found that the SN (i.e., left ACC), ventral AN (left supramarginal gyrus), and left cerebellum were significantly activated in healthy women with a full bladder compared with an empty bladder. rs-fMRI fixation effect analysis revealed significant changes in FC between a full and empty bladder in DMN (i.e., MPFC, cingulate gyrus, inferior lateral temporal gyrus), SMN (i.e., postcentral gyrus), and BGN (i.e., amygdala and caudate nucleus). They suggested that bladder control during bladder filling depends primarily on the functional integration of distributed brain systems. Jarrahi et al. (2015a) found that subliminal stimulation of bladder filling in healthy women can cause significant changes in FC within and between the SN (insula and ACC), SMN, subcortical network (amygdala, hippocampus, and thalamus), and posterior DMN, BGN, cerebellum, and brainstem networks, suggesting that subliminal sensory input may affect mood, emotion, and behavior.

In another fMRI study, Jarrahi et al. (2015b) performed task fMRI of repeated bladder perfusion/withdrawal of saline in four states in healthy women [i.e., empty bladder (warm), empty bladder (cold), 100 ml (warm), and strong desire to void (warm)]. They found that visceral interoception (i.e., bladder distention) in healthy women caused increased activation of brain regions within the SN (anterior insula and ACC), CEN [DLPFC and posterior parietal cortices (PPC)], DMN (MPFC, precuneus/PCC, bilateral parietal lobules, and the inferior temporal gyri), ventral AN [bilateral temporoparietal junction (TPJ)], BGN (bilateral striatum and bilateral amygdala), subcortical network (thalamus and parahippocampa gyri) and cerebellum/brainstem networks. (Jarrahi et al., 2015b). By analyzing the functional network connectivity (FNC), Jarrahi et al. found that bladder filling in all four states could cause an FC decrease between AN and DMN and an FC increase between the DMN and subcortical network. Furthermore, compared with empty bladder (warm), the FC between aDMN and AN in 100 ml (warm) and strong desire to void (warm) state decreases, while the FC between aDMN and BGN increases, as well as DMN and SN. The team indicated that visceral sensation is a dynamic process in which the components interact closely but are separable. In this system, SN (insula, ACC), CEN (DLPFC and posterior parietal cortex), thalamus, and ventral AN (TPJ) provide visceral status monitoring and significance detection, while the DMN (MPFC and IFG), BGN (striatum, and amygdala), SN (insula and ACC), subcortical network (thalamus, hippocampi, and parahippocampal gyrus) and brainstem are more likely involved in the regulation of arousal, motivation, emotion, and action initiation.

### Urgency urinary incontinence

Ketai et al. (2016) found that the activation of some brain regions in the UUI group were greater than in the HC group when the bladder was full, including the interoceptive network (i.e., left insula, ACC and MCC), VAN (i.e., VLPFC, bilateral middle superior temporal and supramarginal gyrus) and posterior DMN (i.e., PCC and Precuneus). They thought the increased desire to void was associated with greater urgency and incontinence. Even before the bladder is full, the FC between MCC and DAN in the UUI group was abnormally stronger than in the HC group. On the contrary, the FC between MCC and VAN in the HC group was stronger than in UUI group. Ketai et al. (2016) suggested that this increased connection to DAN may indicate top-down attentional support for goal-directed (e.g., maintaining continence) behaviors in UUI patients. This is different from HC in that VAN is used for bottom-up attention support.

Nardos et al. (2016) found that UUI patients had significantly abnormal activation of brain regions within the DMN (i.e., inferior parietal lobe), CEN (i.e., inferior and superior frontal gyrus), and SMN (i.e., precentral and postcentral gyrus) compared with HC. They suggested that LUTS is associated with attention, decision making, and primary motor and sensory dysfunction in patients with UUI. Moreover, six typical FC features could predict the severity of UUI, including the connections linked to SN (i.e., dorsal/ventral ACC) and DMN (i.e., AG and ventral medial frontal regions) to SMN areas, between CEN (i.e., superior frontal gyrus) and cerebellum and between SN (i.e., insula) and SMN (i.e., paracentral lobule). They suggest that UUI patients have atypical functional integration between emotional, cognitive, and motor areas that can help distinguish the presence or absence of UUI and predict the severity of symptoms. Clarkson et al. (2018) found that patients with UUI who responded to pelvic floor muscle therapy (PFMT) had significant differences in FC of BGN (caudate nucleus and putamen) and dACC compared with before treatment. They suggest that this variation in FC indicates that the motor processing mechanism may be related to UUI and can be altered by PFMT. Clarkson et al. (2018) found that responders exhibit significant differences in the FC (i.e., between the MPFC and the precuneus, cingulum and postcentral gyrus) from nonresponders at baseline. They suggest that UUI has two subtypes, one primarily caused by abnormalities in brain control (responders) and the other with little to do with brain function (nonresponders). Ketai et al. (2021) found that successful pharmacological treatment of UUI is associated with reduced activation of VAN (bottom-up attention), which may be caused by the drug’s reduction of bladder-afferent impulses. Conversely, the decrease in VAN activation in successful hypnotherapy treatment of UUI may be due to the balancing effect of DAN (top-down attention).

### Overactive bladder

Zuo et al. (2019a) found that in OAB patients, the FC strength within the DMN (i.e., MPFC and PCG), ACG, and MCG was significantly decreased, while the FC strength of middle frontal gyrus (MFG), components of CEN, was significantly increased when compared with healthy controls. They suggested that the reduced FC strength of MPFC, ACG, MCG, and PCG result in inhibition of urine storage and promotes voiding reflex in patients with OAB. In another rs-fMRI study, Zuo et al. (2019a) found that the FC within the SMN (i.e., paracentral lobule), ECN (i.e., both supramarginal gyrus), DAN (i.e., precentral gyrus), dVN (i.e., left cuneus), and LFPN(i.e., superior frontal gyrus) was significantly decreased, as well as the FC between the SMN and the anterior DMN, is reduced in OAB group compared with HC. They believe that these brain networks are related to bladder control, which can perform a series of sensory, motor, emotional, and cognitive processing of incoming signals from the bladder, and evaluate and respond to them in the social environment. These intranetwork and internetwork FC anomalies may affect the OAB.

Our recent study (Pang et al., 2022) showed that the activation of left DLPFC was significantly reduced in OAB patients with a strong desire to void compared with HC and that LUTS improved in OAB patients after 2–4 weeks of sacral neuromodulation (SNM), while the activation of the left DLPFC was restored with no significant difference from HC. We suggest that decreased DLPFC activation in OAB patients releases its inhibition of the voiding reflex, leading to classic OAB symptoms.

### Interstitial cystitis/painful bladder syndrome or urologic chronic pain syndrome

We investigated the FC within the prefrontal cortex (PFC) of patients with IC/BPS versus healthy subjects using the rs-fNIRS. (Pang and Liao, 2021). FC was significantly decreased within the PFC in the IC/BPS group, whether with an empty bladder or a strong desire to void. Moreover, compared with the empty bladder state (BA9,10, and 46; 18 edges), the FC decreased in a wider range during the strong desire to void (BA9,10,45, and 46; 28 edges). Edge stands for FC between two brain regions. We suggest that the abnormal reduction of FC within the PFC in IC/BPS patients may cause the release of inhibition of the PFC on the voiding reflex, causing LUTS. Kilpatrick et al. (2014) found significant changes in the frequency distribution of visceral sensation (insula), somatosensory (postcentral gyrus), and motor area (anterior paracentral lobule and SMA) in IC/BPS patients compared with HC. They suggest that sensory and motor dysfunction in IC/BPS patients is a pathological mechanism. Kilpatrick et al. (2014) found that the insula and SMA were enhanced with FC in the midbrain (red nucleus) and cerebellum, and they suggested that it is also a manifestation of IC/BPS pathology. Martucci et al. (2015) found that FC of DMN was significantly reduced to the PCC and left precuneus in women with urologic chronic pain syndrome (UCPPS) compared with HC. They suggested that DMN dissociation occurs in UCPPS patients and that pain and emotional regulation may be related to the self-referential thinking and introspective neural processes responsible for DMN.

### Neurogenic overactive bladder

Gao et al. (2021) found that PFC and ACC were activated in neurogenic detrusor overactivity (NDO) patients with tethered cord syndrome compared with the HC group. Moreover, compared with HC, FC analysis showed increased FC between different regions within the PFC and decreased FC between the PFC and other brain regions in NDO patients. They suggest that decreased FC in PFC and other bladder control-related brain regions may be related to reduced PFC decision-making function, which may affect bladder control.

### Nocturnal enuresis

Jiang et al. (2018) found that the degree centrality values of posterior cerebellar lobe, ACC, MPFC, and the left superior temporal gyrus in children with nocturnal enuresis (NE) were significantly decreased in HC, suggesting that these brain regions may be associated with NE. Lei et al. (2012) used rs-fMRI to study changes in spontaneous brain activity in children with primary monosymptomatic NE. They found significant differences in amplitude of low frequency fluctuation (ALFF) or ReHo in the left inferior frontal gyrus/LPFC and MPFC (Brodmann area, BA10) compared with HC, suggesting that abnormalities in the inferior frontal gyrus/LPFC and MPFC may affect children’s urination decision-making ability.

## Discussion

Our review has two novel hypotheses. First, we summarized studies on the activation changes of brain regions during the micturition phase, illuminating possible brain circuits during micturition. Second, we summarized brain FC and brain network studies related to bladder control, providing new information on the existing bladder control model proposed by Griffiths et al. (2015a) (de Groat et al., 2015) in 2015.

### Brain circuits associated with micturition

Only six previous brain functional imaging studies on the micturition phase have been conducted, far fewer than those on the storage phase. To better explain brain activity during voiding, we summarized the six studies (Table 1) and found that the frequency of activation of brain regions during micturition was five times more for the LPFC/inferior frontal gyrus and pons; three times more for the PAG, hypothalamus, basal ganglia, and ACC; twice for the postcentral gyrus, thalamus, insula, superior frontal gyrus, cerebellar, and SMA.

In short, the LPFC is activated both during storage and urination, while the MPFC is deactivated during storage but activated during micturition. Pons is activated only during micturition, while the PAG, ACC, insula, thalamus, hypothalamus, basal ganglia, and postcentral gyrus are activated in both phases. We believe that, similar to urine storage, micturition also requires DLPFC activity for executive function (i.e., organization of input from different sensory modes (e.g., bladder sensation and vision), maintaining attention, monitoring information (e.g., surroundings and social etiquette) in working memory, and coordinating goal-directed (voiding) behavior. (Teffer and Semendeferi, 2012).

Given that MPFC activation is critical for the representation of reward- and value-based decisions, (Hiser and Koenigs, 2018), we suggest that decisions about voiding are driven by MPFC activation at the onset of micturition. Manohar et al. (2017) found that LC and MPFC activation in rats occurred synchronously about 20 s before urination, presenting consistent *θ* oscillations, which they suggested shifted the rats from ongoing behaviors unrelated to urination to initiation of specific urination behaviors in order to urinate in appropriate conditions. Although PAG is activated in both phases, the degree of activation may be different. (Griffiths, 2015a). We considered that the activation of MPFC may further increase the activity of PAG to exceed a certain threshold, activating the pons, which transmits the void impulse to the spinal cord and controls the coordinated movement of detrusor muscle and urethral sphincter to achieve voiding.

The ACC, insula, thalamus, hypothalamus, basal ganglia, and postcentral gyrus have been reported to be activated in both phases, and we believe that these brain regions play a similar role in urine storage and urination. For example, bladder sensation is uploaded to the insula *via* the thalamus, and the insula and ACC, as parts of the SN, are responsible for sensing and encoding the desire to void. The postcentral gyrus may be involved in somatosensory perception as part of the SMN. Yamamoto et al. (2005) analyzed three patients with hypothalamic compression due to pituitary adenoma and found that hypothalamic lesions could lead to DO during urine storage and detrusor insufficiency during urination, suggesting that the hypothalamus plays a role in both phases. The caudate nucleus, a component of basal ganglia (BG), was thought to has an inhibitory effect on micturition reflex. (Seseke et al., 2008; Gao et al., 2015). Electrical stimulation applied to the caudate nucleus can cause inhibition of spontaneous bladder contraction in cats. (Yamamoto et al., 2009).

### Brain FC and networks related to bladder control: An extended working model

Most previous studies focused on the activation/inactivation of a specific brain region related to bladder control. Brain regions do not work independently, and the separation, integration, communication, and cooperation between regions are important mechanisms of brain-bladder control, which depends on the brain FC or network. By summarizing the brain FC network articles related to bladder control over the past 20 years, we found five studies on HC, four on UUI, two on OAB, three on IC/BPS or UCPPS, two on NE, and one on NDO patients with tethered cord syndrome. These studies all explored brain FC network changes during the urine storage phase using fMRI and fNIRS, without observing the micturition phase.

**FIGURE 1:**
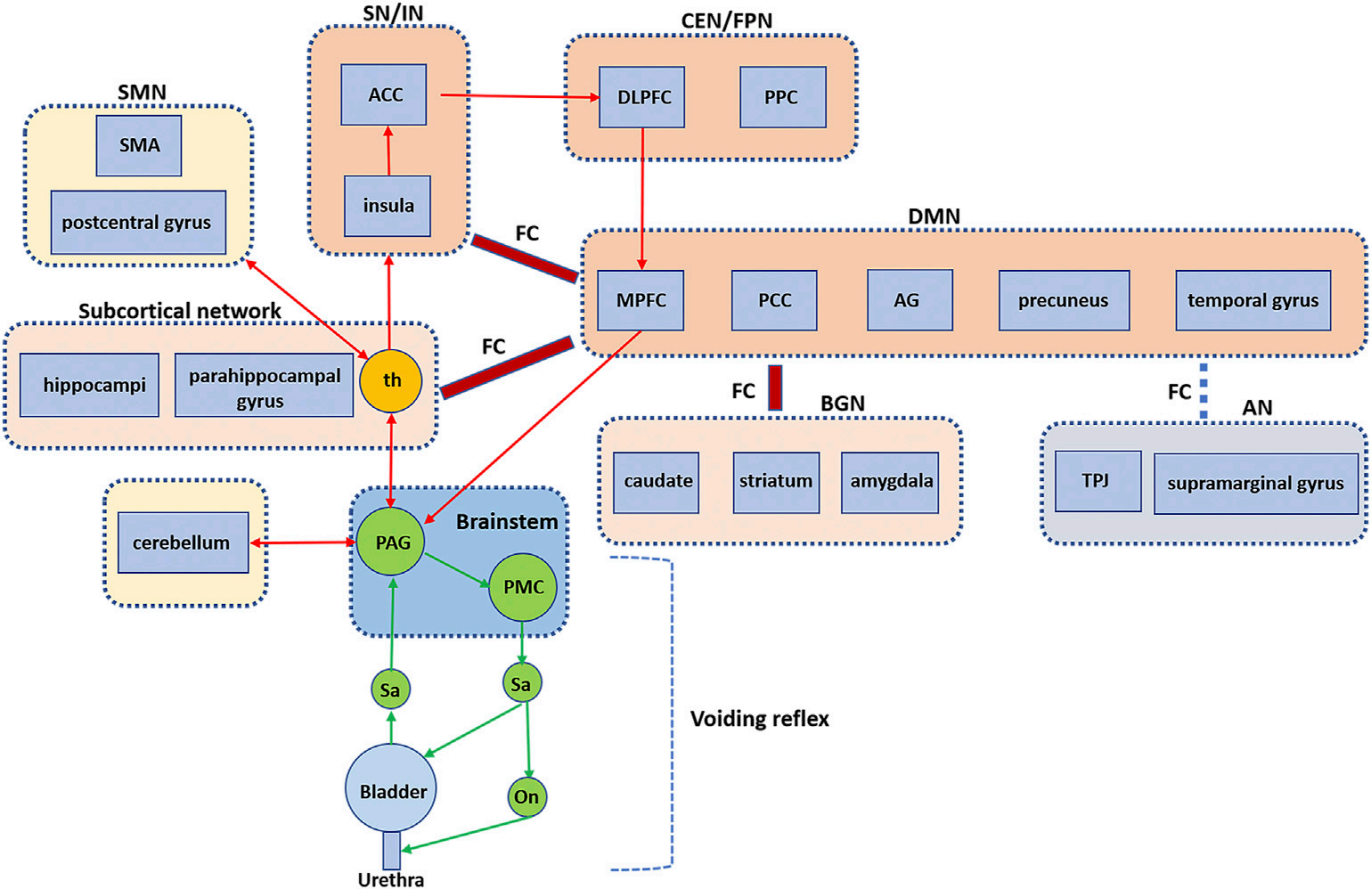
Brain FC and networks related to bladder control. An extended working mode showing the voiding reflex and the brain circuits, especially the interaction between brain regions and networks. FC, functional connectivity; PAG, periaqueductal gray; PMC, pontine micturition center; th, thalamus; SMA, supplementary motor area; SMN, sensorimotor network; ACC, anterior cingulate cortex; SN, salience network; IN, interoceptive network; DLPFC, dorsolateral prefrontal cortex; PPC, posterior parietal cortices; CEN, central executive network; FPN, frontoparietal network; MPFC = medial prefrontal cortex; PCC, posterior cingulate cortex; AG, angular gyrus; DMN, default mode network; BGN, basal ganglia network; AN, attentional network; TPJ, temporoparietal junction. (Extended based on working model by de Groat et al., 2015).

The results of the five studies on HC showed increased activity within certain brain networks during urine storage in healthy patients, including the ventral AN (bilateral TPJ and supramarginal gyrus), CEN/FPN (DLPFC and posterior parietal cortices), SN/IN (insula and ACC), DMN (superior frontal gyrus, PCG, precuneus, parietal lobules, temporal gyrus, and AG), SMN (postcentral gyrus), VN (superior occipital gyrus, middle occipital gyrus, and cuneus), BGN (caudate nucleus, striatum, and amygdala), subcortical network (thalamus, hippocampus, and parahippocampal gyrus), cerebellum, and brainstem. Changes in interactions between networks and bladder distention could cause decreases in FC between AN and DMN and increases in FC between the DMN and the subcortical network. Moreover, the repeated perfusion/withdrawal task with prefilling (100 ml or strong desire to void) can cause a decrease in FC between aDMN and AN and an increase in FC between aDMN and BGN and between DMN and SN, compared with the task with an empty bladder. Based on these results, we extend the working model proposed by Griffiths et al. (Griffiths, 2015a; de Groat et al., 2015) to include the specific brain regions and networks involved in bladder control and the interactions between networks (Figure 1). We performed rs-fMRI imaging of 20 healthy subjects with empty and full bladder. We identified seven known resting state networks using independent component analysis method, including SN, DMN, CEN, dAN, SMN, VN, and cerebellum network (CN), as shown in our previous article. (Pang et al., 2021). This will help us understand the composition of these networks.

Under normal circumstances, the physiological filling of the bladder is a slow process from subthreshold (no sensation) to upper threshold (e.g., first sensation of bladder filling, first desire to void, normal desire to void, strong desire to void, urgency desire to void, and pain). The perception of bladder filling/distention is a type of interoception, which is defined as all sensations produced in the body, (Craig, 2003), including pressure, fullness, and pain of the visceral organs. (Cameron, 2001). The signal of bladder dilation is uploaded to the thalamus via PAG and then relayed to the insula and the interoceptive afferent cortex, (Blok et al., 1997; Craig, 2003), which encodes the degree of bladder filling and forms the interoceptive awareness of bladder sensation. (Craig, 2003; Griffiths and Tadic, 2008). The ACC and insula are part of the SN, which has extensive coactivation and plays a role in integrating internal and external information. (Torta et al., 2013; Jarrahi et al., 2015b). The SN is responsible for locating and detecting associated stimuli. (Seeley et al., 2007). Previous studies have shown that ACC can initiate autonomic responses and prompt goal-directed behaviors such as withholding urine or void. (Craig, 2003). As the bladder continues to dilate, SN becomes more aware of this interoception, forming a gradually increased desire to void, which may activate another network (i.e., CEN). (Jarrahi et al., 2015a). CEN consists of the DLPFC and the lateral posterior parietal cortex (PCC). (Chan et al., 2016).

Anticipation and attention to visceral sensation and pain depend on DLPFC. (Aziz et al., 2000). DLPFC can maintain attention, organize input from different sensory modes (e.g., bladder sensation), monitor environment and social etiquette in working memory, judge whether it is appropriate to void, and decide on goal-directed (withholding/voiding) behavior. (Teffer and Semendeferi, 2012; Chan et al., 2016). In other words, CEN allows us to make behavioral choices by continually paying attention to salient stimuli, balancing the changing environment against the changing demands of interoception. (Seeley et al., 2007).

MPFC, as part of DMN, is usually reported to be activated during micturition (Nour et al., 2000; Shy et al., 2014) and deactivated during urine storage. (Blok et al., 1997; Blok et al., 1998; Griffiths et al., 2007; Griffiths et al., 2009). In contrast, Jarrahi et al. (Jarrahi et al., 2015a) found activation of brain regions (i.e., MPFC, the precuneus/PCC, bilateral parietal lobules, and the inferior temporal gyri) within the DMN. We found increased ReHo in the brain region within DMN (i.e., left temporal gyrus and left AG) and increased FC within the DMN (i.e., superior frontal gyrus, PCG, and AG) when the desire to void was strong, compared with an empty bladder. These inconsistent results may suggest that DMN activity is isolated or dynamic during urine storage, which is worthy of further exploration. Previous studies have shown that stimulation of PCG can interrupt urination when a cat’s bladder is rapidly filled. (Gjone, 1966). AG is responsible for episodic memory, which allows recall of past experiences. (Bonnici et al., 2018). DMN has been reported to be activated at rest (i.e., no significant external stimulus task) (Raichle, 2015) but has also been reported to be active in introverted cognitive activities such as self-reference, self-reflection, social functioning, and physiological processes. (Nardos et al., 2014; Raichle, 2015). The DMN is thought to be involved in physiological processes such as bladder control, possibly through interoception mechanisms or self-reflection. (Nardos et al., 2014). The DMN may be able to support internal psychology by simulating the dynamic psychological changes of past experiences. (Nardos et al., 2016; Bonnici et al., 2018). Animal studies have shown that MPFC is the main source of cortical-PAG projection and that MPFC is involved in autonomic and emotional regulation of external stimuli through direct connection with PAG. (Hardy and Leichnetz, 1981). The deactivation of MPFC may reduce MPFC input to PAG, stabilizing PAG, inhibiting the voiding reflex, and maintaining continence. In addition, mPFC projections to other regions, particularly the hypothalamus and extended amygdala, play an equally plausible role to bladder control. (Pajolla et al., 2001).

Moreover, other networks are involved in bladder control during urinary storage, including the VAN (bilateral TPJ and supramarginal gyrus) response to unexpected events (bottom-up attention). (Vossel et al., 2014). The VAN was reported to be activated when the bladder was full, which is also easy to understand because visceral changes are unexpected events, inside-out processes that can attract bottom-up attention. (Vossel et al., 2014; Jarrahi et al., 2015b). The subcortical network (thalamus, hippocampi, and parahippocampal gyrus), which overlaps with limbic correlates, has been reported to be involved in the intersensory processing of smell, taste, and hunger responses. (Laird et al., 2011). The thalamus is responsible for motor/sensory relay and consciousness regulation. (Smitha et al., 2017). The subcortical network is also similar to neural circuit 3 proposed by Griffiths et al. (2015a) (de Groat et al., 2015). Activation of the subcortical network may be involved in unconscious monitoring of sensory information in the bladder when bladder volume is small, and there is little sensation. (Griffiths, 2015a; de Groat et al., 2015).

The BGN (caudate nucleus, striatum, and amygdala) showed increased activity during urine storage, supporting the hypothesis that BGN inhibits the voiding reflex (Seseke et al., 2008; Gao et al., 2015) and that electrical stimulation of the caudate nucleus results in inhibition of spontaneous bladder contraction in cats. (Yamamoto et al., 2009). Previous studies have shown that the caudate nucleus is involved in motor processing, process learning, and control of motor inhibition, while the putamen is responsible for motor regulation. (Smitha et al., 2017). BGN is responsible for the process of motor area control, emotion, and cognition. BGN engage in goal-directed behavior that requires movement. (Smitha et al., 2017). VN located in the visual cortex and is divided into dorsal VN and ventral VN. The dorsal VN processes information about the position of objects and adjusts visual controls for skilled movements. (Migliaccio et al., 2016). Although significant activation of the occipital lobe (i.e., VN) has been reported many times, (Seseke et al., 2006; Griffiths and Tadic, 2008; Seseke et al., 2008; Ketai et al., 2016; Nardos et al., 2016; Zuo et al., 2019a), its function remains unclear and needs to be elucidated in further studies.

### Brain FC network changes in patients with LUTD

According to our review, 12 brain FC/network studies have been conducted in patients with LUTD (four studies on UUI, two on OAB, three on IC/BPS or UCPPS, two on NE, and one on NDO patients with tethered cord syndrome). A common feature of these disorders is that LUTS are often present in the urinary storage phase (e.g., urinary frequency and urgency, UUI, pain/discomfort of bladder and pelvis).

Compared with HC, UUI patients showed abnormal activation of brain regions within the SN, VAN, DMN, CEN, and SMN. (Ketai et al., 2016; Nardos et al., 2016). The FC between MCC and DAN in the UUI group was stronger than in the HC group. (Ketai et al., 2016). Moreover, six typical FC features could predict the severity of UUI, including the connections between SN and DMN to SMN and between CEN and SN and SMN. (Nardos et al., 2016). Some scholars have explored the central mechanism of effective treatment of UUI, finding that UUI patients who responded to PFMT had significant differences in FC of BGN and dACC compared with before treatment. (Clarkson et al., 2018). UUI successfully treated with pharmacotherapy is associated with reduced activation of VAN, while the decrease in VAN activation in the successful treatment of UUI using hypnotherapy may be due to the balancing effect of DAN. (Ketai et al., 2021). We found that the central mechanism of SNM treatment for OAB may be restoration of the activation of the left DLPFC to a near-normal level. (Pang et al., 2022). Compared with HC patients, OAB patients showed that the FC strength within the DMN, ACG, and MCG was significantly decreased, while the FC strength within the CEN was significantly increased. (Zuo et al., 2019b). The FC within the SMN, DAN, dVN, and LFPN was significantly decreased, as was the FC between the SMN and the anterior DMN. (Zuo et al., 2019a).

Compared with HC, the IC/BPS patients showed significantly decreased FC within the PFC, whether with an empty bladder or a strong desire to void. (Pang and Liao, 2021). IC/BPS patients showed significant changes in the frequency distribution of SN and SMN and enhanced FC between the insula, SMA, midbrain, and cerebellum. (Kilpatrick et al., 2014). The FC of the DMN was significantly reduced to the PCC and left precuneus in women with UCPPS. (Martucci et al., 2015). Compared with HC patients, NDO patients with tethered cord syndrome showed deactivation in the PFC and ACC, increased FC within the PFC, and decreased FC between the PFC and other brain regions. (Gao and Liao, 2021). Compared with HC patients, the NE patients showed a decreased degree centrality of the posterior cerebellar lobe, ACC, MPFC, and left superior temporal gyrus. (Jiang et al., 2018). NE patients showed significant differences in ALFF or ReHo in the left inferior frontal gyrus/LPFC and MPFC (Brodmann area, BA10). (Lei et al., 2012). Problems with collaboration and communication within networks, as well as functional integration between networks, may be the central mechanism of LUTD. Due to differences in experimental paradigms and analytical methods, precise interpretation of these results is difficult.

In summary, bladder control involves complex neural networks, and neural control related to micturition control is still an unstudied area, with many unexplored areas. For example, there have been no studies of the: 1) Brain FC network in healthy subjects and patients (e.g., UAB) during micturition; 2) Dynamic changes of the DMN network during urine storage and micturition in healthy subjects and patients and the relationship and interaction between MPFC and activities of other brain regions within DMN; 3) Dynamic changes and roles of regions such as the VN, SMN, cerebellum, subcortical networks in bladder control; 4) Brain targeted therapy based on abnormal brain activity or FC; and 5) Other methods that can regulate the brain network for LUTD treatment. For instance, Ketai et al. found that the activation of VAN in UUI was abnormally higher than that in HC (Ketai et al., 2016) and improved LUTS in UUI through hypnotherapy. The possible mechanism was to balance the abnormally elevated VAN in UUI patients by upregulating DAN and, ultimately, reduce VAN activity in UUI. (Ketai et al., 2016).

## References

[B1] Ackermann R. F. Finch D. M. Babb T. L. Engel J. Jr (1984). Increased glucose metabolism during long-duration recurrent inhibition of hippocampal pyramidal cells. J. Neurosci. 4, 251–264. 10.1523/jneurosci.04-01-00251.1984 6693941 PMC6564752

[B2] Andrew J. Nathan P. W. (1964). Lesions on the anterior frontal lobes and disturbances of micturition and defaecation. Brain 87, 233–262. 10.1093/brain/87.2.233 14188274

[B3] Aziz Q. Thompson D. G. Ng V. W. Hamdy S. Sarkar S. Brammer M. J. (2000). Cortical processing of human somatic and visceral sensation. J. Neurosci. 20, 2657–2663. 10.1523/jneurosci.20-07-02657.2000 10729346 PMC6772246

[B4] Batini C. Benedetti F. Buisseret-Delmas C. Montarolo P. G. Strata P. (1984). Metabolic activity of intracerebellar nuclei in the rat: Effects of inferior olive inactivation. Exp. Brain Res. 54, 259–265. 10.1007/BF00236225 6723846

[B5] Blok B. F. Sturms L. M. Holstege G. (1998). Brain activation during micturition in women. Brain 121 (Pt 11), 2033–2042. 10.1093/brain/121.11.2033 9827764

[B6] Blok B. F. Willemsen A. T. Holstege G. (1997). A PET study on brain control of micturition in humans. Brain 120 (Pt 1), 111–121. 10.1093/brain/120.1.111 9055802

[B7] Bonnici H. M. Cheke L. G. Green D. A. E. FitzGerald T. Simons J. S. (2018). Specifying a causal role for angular gyrus in autobiographical memory. J. Neurosci. 38, 10438–10443. 10.1523/JNEUROSCI.1239-18.2018 30355636 PMC6284111

[B8] Cameron O. G. (2001). Interoception: The inside story--a model for psychosomatic processes. Psychosom. Med. 63, 697–710. 10.1097/00006842-200109000-00001 11573016

[B9] Chan J. S. Wang Y. Yan J. H. Chen H. (2016). Developmental implications of children's brain networks and learning. Rev. Neurosci. 27, 713–727. 10.1515/revneuro-2016-0007 27362958

[B10] Clarkson B. D. Karim H. T. Griffiths D. J. Resnick N. M. (2018). Functional connectivity of the brain in older women with urgency urinary incontinence. Neurourol. Urodyn. 37, 2763–2775. 10.1002/nau.23766 30054930 PMC6469490

[B11] Craig A. D. (2003). Interoception: The sense of the physiological condition of the body. Curr. Opin. Neurobiol. 13, 500–505. 10.1016/s0959-4388(03)00090-4 12965300

[B12] de Groat W. C. Griffiths D. Yoshimura N. (2015). Neural control of the lower urinary tract. Compr. Physiol. 5, 327–396. 10.1002/cphy.c130056 25589273 PMC4480926

[B13] Duffau H. Capelle L. (2005). Incontinence after brain glioma surgery: New insights into the cortical control of micturition and continence. Case report. J. Neurosurg. 102, 148–151. 10.3171/jns.2005.102.1.0148 15658106

[B14] Fowler C. J. Griffiths D. de Groat W. C. (2008). The neural control of micturition. Nat. Rev. Neurosci. 9, 453–466. 10.1038/nrn2401 18490916 PMC2897743

[B15] Fox M. D. Raichle M. E. (2007). Spontaneous fluctuations in brain activity observed with functional magnetic resonance imaging. Nat. Rev. Neurosci. 8, 700–711. 10.1038/nrn2201 17704812

[B16] Fukuyama H. Matsuzaki S. Ouchi Y. YamaucHi H. Nagahama Y. Kimura J. (1996). Neural control of micturition in man examined with single photon emission computed tomography using 99mTc-HMPAO. Neuroreport 7, 3009–3012. 10.1097/00001756-199611250-00042 9116229

[B17] Gao Y. Liao L. Blok B. F. M. (2015). A resting-state functional MRI study on central control of storage: Brain response provoked by strong desire to void. Int. Urol. Nephrol. 47, 927–935. 10.1007/s11255-015-0978-0 25917482

[B18] Gao Y. Liao L. (2021). Regional activity and functional connectivity in brain networks associated with urinary bladder filling in patients with tethered cord syndrome. Int. Urol. Nephrol. 53, 1805–1812. 10.1007/s11255-021-02880-0 34152553

[B19] Gjone R. (1966). Excitatory and inhibitory bladder responses to stimulation of 'limbic', diencephalic and mesencephalic structures in the cat. Acta Physiol. Scand. 66, 91–102. 10.1111/j.1748-1716.1966.tb03171.x 5327579

[B20] Griffiths D. Derbyshire S. Stenger A. Resnick N. (2005). Brain control of normal and overactive bladder. J. Urol. 174, 1862–1867. 10.1097/01.ju.0000177450.34451.97 16217325

[B21] Griffiths D. (2015). Neural control of micturition in humans: A working model. Nat. Rev. Urol. 12, 695–705. 10.1038/nrurol.2015.266 26620610

[B22] Griffiths D. (2015). Functional imaging of structures involved in neural control of the lower urinary tract. Handb. Clin. Neurol. 130, 121–133. 10.1016/B978-0-444-63247-0.00007-9 26003241

[B23] Griffiths D. Tadic S. D. (2008). Bladder control, urgency, and urge incontinence: Evidence from functional brain imaging. Neurourol. Urodyn. 27, 466–474. 10.1002/nau.20549 18092336

[B24] Griffiths D. Tadic S. D. Schaefer W. Resnick N. M. (2007). Cerebral control of the bladder in normal and urge-incontinent women. Neuroimage 37, 1–7. 10.1016/j.neuroimage.2007.04.061 17574871 PMC2075467

[B25] Griffiths D. J. Tadic S. D. Schaefer W. Resnick N. M. (2009). Cerebral control of the lower urinary tract: How age-related changes might predispose to urge incontinence. Neuroimage 47, 981–986. 10.1016/j.neuroimage.2009.04.087 19427909 PMC2719686

[B26] Hardy S. G. Leichnetz G. R. (1981). Cortical projections to the periaqueductal gray in the monkey: A retrograde and orthograde horseradish peroxidase study. Neurosci. Lett. 22, 97–101. 10.1016/0304-3940(81)90070-7 6164962

[B27] Hiser J. Koenigs M. (2018). The multifaceted role of the ventromedial prefrontal cortex in emotion, decision making, social cognition, and psychopathology. Biol. Psychiatry 83, 638–647. 10.1016/j.biopsych.2017.10.030 29275839 PMC5862740

[B28] Jarrahi B. Mantini D. Balsters J. H. Michels L. Kessler T. M. Mehnert U. (2015a). Differential functional brain network connectivity during visceral interoception as revealed by independent component analysis of fMRI TIME-series. Hum. Brain Mapp. 36, 4438–4468. 10.1002/hbm.22929 26249369 PMC6869370

[B29] Jarrahi B. Mantini D. Mehnert U. Kollias S. (2015b). Exploring influence of subliminal interoception on whole-brain functional network connectivity dynamics. Annu. Int. Conf. IEEE Eng. Med. Biol. Soc. 2015, 670–674. 10.1109/EMBC.2015.7318451 26736351

[B30] Jiang K. Ding L. Li H. Shen H. Zheng A. Zhao F. (2018). Degree centrality and voxel-mirrored homotopic connectivity in children with nocturnal enuresis: A functional MRI study. Neurol. India 66, 1359–1364. 10.4103/0028-3886.241334 30233003

[B31] Jobsis F. F. (1977). Noninvasive, infrared monitoring of cerebral and myocardial oxygen sufficiency and circulatory parameters. Sci. (New York, NY) 198, 1264–1267. 10.1126/science.929199 929199

[B32] Ketai L. H. Komesu Y. M. Dodd A. B. Rogers R. G. Ling J. M. Mayer A. R. (2016). Urgency urinary incontinence and the interoceptive network: A functional magnetic resonance imaging study. Am. J. Obstet. Gynecol. 215, e441–449. 10.1016/j.ajog.2016.04.056 PMC5045785 27173081

[B33] Ketai L. H. Komesu Y. M. Schrader R. M. Rogers R. G. Sapien R. E. Dodd A. B. (2021). Mind-body (hypnotherapy) treatment of women with urgency urinary incontinence: Changes in brain attentional networks. Am. J. Obstet. Gynecol. 224, 498.e1–498.e10. 10.1016/j.ajog.2020.10.041 33122028 PMC10739935

[B34] Kilpatrick L. A. Kutch J. J. Tillisch K. Naliboff B. D. Labus J. S. Jiang Z. (2014). Alterations in resting state oscillations and connectivity in sensory and motor networks in women with interstitial cystitis/painful bladder syndrome. J. Urol. 192, 947–955. 10.1016/j.juro.2014.03.093 24681331 PMC4432915

[B35] Kitta T. Mitsui T. Kanno Y. Chiba H. Moriya K. Shinohara N. (2015). Brain-bladder control network: The unsolved 21st century urological mystery. Int. J. Urol. 22, 342–348. 10.1111/iju.12721 25693685

[B36] Krhut J. Tintera J. Holy P. Zachoval R. Zvara P. (2012). A preliminary report on the use of functional magnetic resonance imaging with simultaneous urodynamics to record brain activity during micturition. J. Urol. 188, 474–479. 10.1016/j.juro.2012.04.004 22698619

[B37] Kuhtz-Buschbeck J. P. van der Horst C. Pott C. Wolff S. Nabavi A. Jansen O. (2005). Cortical representation of the urge to void: A functional magnetic resonance imaging study. J. Urol. 174, 1477–1481. 10.1097/01.ju.0000173007.84102.7c 16145475

[B38] Laird A. R. Fox P. M. Eickhoff S. B. Turner J. A. Ray K. L. McKay D. R. (2011). Behavioral interpretations of intrinsic connectivity networks. J. Cogn. Neurosci. 23, 4022–4037. 10.1162/jocn_a_00077 21671731 PMC3690655

[B39] Lei D. Ma J. Du X. Shen G. Tian M. Li G. (2012). Spontaneous brain activity changes in children with primary monosymptomatic nocturnal enuresis: A resting-state fMRI study. Neurourol. Urodyn. 31, 99–104. 10.1002/nau.21205 22038619

[B40] Manohar A. Curtis A. L. Zderic S. A. Valentino R. J. (2017). Brainstem network dynamics underlying the encoding of bladder information. Elife 6, e29917. 10.7554/eLife.29917 29199948 PMC5714501

[B41] Martucci K. T. Shirer W. R. Bagarinao E. Johnson K. A. Farmer M. A. Labus J. S. (2015). The posterior medial cortex in urologic chronic pelvic pain syndrome: Detachment from default mode network-a resting-state study from the MAPP research network. Pain 156, 1755–1764. 10.1097/j.pain.0000000000000238 26010458 PMC4545714

[B42] Matsumoto S. Ishikawa A. Matsumoto S. Homma Y. (2011). Brain response provoked by different bladder volumes: A near infrared spectroscopy study. Neurourol. Urodyn. 30, 529–535. 10.1002/nau.21016 21284027

[B43] Migliaccio R. Gallea C. Kas A. Perlbarg V. Samri D. Trotta L. (2016). Functional connectivity of ventral and dorsal visual streams in posterior cortical atrophy. J. Alzheimers Dis. 51, 1119–1130. 10.3233/JAD-150934 26923019

[B44] Munoz M. F. Puebla M. Figueroa X. F. (2015). Control of the neurovascular coupling by nitric oxide-dependent regulation of astrocytic Ca(2+) signaling. Front. Cell. Neurosci. 9, 59. 10.3389/fncel.2015.00059 25805969 PMC4354411

[B45] Nardos R. Gregory W. T. Krisky C. Newell A. Nardos B. Schlaggar B. (2014). Examining mechanisms of brain control of bladder function with resting state functional connectivity MRI. Neurourol. Urodyn. 33, 493–501. 10.1002/nau.22458 23908139

[B46] Nardos R. Karstens L. Carpenter S. Aykes K. Krisky C. Stevens C. (2016). Abnormal functional connectivity in women with urgency urinary incontinence: Can we predict disease presence and severity in individual women using Rs-fcMRI. Neurourol. Urodyn. 35, 564–573. 10.1002/nau.22767 25933352

[B47] Nour S. Svarer C. Kristensen J. K. Paulson O. B. Law I. (2000). Cerebral activation during micturition in normal men. Brain 123 (Pt 4), 781–789. 10.1093/brain/123.4.781 10734009

[B48] Pajolla G. P. Crippa G. E. Corrêa S. A. Moreira K. B. Tavares R. F. Corrêa F. M. (2001). The lateral hypothalamus is involved in the pathway mediating the hypotensive response to cingulate cortex-cholinergic stimulation. Cell. Mol. Neurobiol. 21, 341–356. 10.1023/a:1012650021137 11775065 PMC11533853

[B49] Pang D. Gao Y. Liao L. (2021). Responses of functional brain networks to bladder control in healthy adults: A study using regional homogeneity combined with independent component analysis methods. Int. Urol. Nephrol. 53, 883–891. 10.1007/s11255-020-02742-1 33523398

[B50] Pang D. Gao Y. Liao L. Ying X. (2020). Brain functional network alterations caused by a strong desire to void in healthy adults: A graph theory analysis study. Neurourol. Urodyn. 39, 1966–1976. 10.1002/nau.24445 32806881

[B51] Pang D. Liao L. (2021). Abnormal functional connectivity within the prefrontal cortex in interstitial cystitis/bladder pain syndrome (IC/BPS): A pilot study using resting state functional near-infrared spectroscopy (rs-fNIRS). Neurourol. Urodyn. 40, 1634–1642. 10.1002/nau.24729 34130350

[B52] Pang D. Liao L. Chen G. Wang Y. (2022). Sacral neuromodulation improves abnormal prefrontal brain activity in patients with overactive bladder: A possible central mechanism. J. Urol. 207, 1256–1267. 35072489 10.1097/JU.0000000000002445

[B53] Raichle M. E. MacLeod A. M. Snyder A. Z. Powers W. J. Gusnard D. A. Shulman G. L. (2001). A default mode of brain function. Proc. Natl. Acad. Sci. U. S. A. 98, 676–682. 10.1073/pnas.98.2.676 11209064 PMC14647

[B54] Raichle M. E. (2015). The brain's default mode network. Annu. Rev. Neurosci. 38, 433–447. 10.1146/annurev-neuro-071013-014030 25938726

[B55] Sakakibara R. Hattori T. Yasuda K. Yamanishi T. (1996). Micturitional disturbance after acute hemispheric stroke: Analysis of the lesion site by CT and MRI. J. Neurol. Sci. 137, 47–56. 10.1016/0022-510x(95)00322-s 9120487

[B56] Sakakibara R. Tsunoyama K. Takahashi O. Sugiyama M. Kishi M. Ogawa E. (2010). Real-time measurement of oxyhemoglobin concentration changes in the frontal micturition area: An fNIRS study. Neurourol. Urodyn. 29, 757–764. 10.1002/nau.20815 20583001

[B57] Seeley W. W. Menon V. Schatzberg A. F. Keller J. Glover G. H. Kenna H. (2007). Dissociable intrinsic connectivity networks for salience processing and executive control. J. Neurosci. 27, 2349–2356. 10.1523/JNEUROSCI.5587-06.2007 17329432 PMC2680293

[B58] Seseke S. Baudewig J. Kallenberg K. Ringert R. H. Seseke F. Dechent P. (2008). Gender differences in voluntary micturition control: An fMRI study. Neuroimage 43, 183–191. 10.1016/j.neuroimage.2008.07.044 18721889

[B59] Seseke S. Baudewig J. Kallenberg K. Ringert R. H. Seseke F. Dechent P. (2006). Voluntary pelvic floor muscle control--an fMRI study. Neuroimage 31, 1399–1407. 10.1016/j.neuroimage.2006.02.012 16574434

[B60] Shy M. Fung S. Boone T. B. Karmonik C. Fletcher S. G. Khavari R. (2014). Functional magnetic resonance imaging during urodynamic testing identifies brain structures initiating micturition. J. Urol. 192, 1149–1154. 10.1016/j.juro.2014.04.090 24769029 PMC5485249

[B61] Smitha K. A. Akhil Raja K. Arun K. M. Rajesh P. G. Thomas B. Kapilamoorthy T. R. (2017). Resting state fMRI: A review on methods in resting state connectivity analysis and resting state networks. Neuroradiol. J. 30, 305–317. 10.1177/1971400917697342 28353416 PMC5524274

[B62] Stackhouse T. L. Mishra A. (2021). Neurovascular coupling in development and disease: Focus on astrocytes. Front. Cell Dev. Biol. 9, 702832. 10.3389/fcell.2021.702832 34327206 PMC8313501

[B63] Tadic S. D. Griffiths D. Schaefer W. Cheng C. I. Resnick N. M. (2010). Brain activity measured by functional magnetic resonance imaging is related to patient reported urgency urinary incontinence severity. J. Urol. 183, 221–228. 10.1016/j.juro.2009.08.155 19913803 PMC2869077

[B64] Teffer K. Semendeferi K. (2012). Human prefrontal cortex: Evolution, development, and pathology. Prog. Brain Res. 195, 191–218. 10.1016/B978-0-444-53860-4.00009-X 22230628

[B65] Torta D. M. Costa T. Duca S. Fox P. T. Cauda F. (2013). Parcellation of the cingulate cortex at rest and during tasks: A meta-analytic clustering and experimental study. Front. Hum. Neurosci. 7, 275. 10.3389/fnhum.2013.00275 23785324 PMC3682391

[B66] Vossel S. Geng J. J. Fink G. R. (2014). Dorsal and ventral attention systems: Distinct neural circuits but collaborative roles. Neuroscientist 20, 150–159. 10.1177/1073858413494269 23835449 PMC4107817

[B67] Wager T. D. van Ast V. A. Hughes B. L. Davidson M. L. Lindquist M. A. Ochsner K. N. (2009). Brain mediators of cardiovascular responses to social threat, part II: Prefrontal-subcortical pathways and relationship with anxiety. Neuroimage 47, 836–851. 10.1016/j.neuroimage.2009.05.044 19465135 PMC4169880

[B68] Yamamoto T. Sakakibara R. Nakazawa K. Uchiyama T. Shimizu E. Hattori T. (2009). Effects of electrical stimulation of the striatum on bladder activity in cats. Neurourol. Urodyn. 28, 549–554. 10.1002/nau.20682 19214990

[B69] Yamamoto T. Sakakibara R. Uchiyama T. Liu Z. ITo T. Yamanishi T. (2005). Lower urinary tract function in patients with pituitary adenoma compressing hypothalamus. J. Neurol. Neurosurg. Psychiatry 76, 390–394. 10.1136/jnnp.2004.044644 15716534 PMC1739555

[B70] Yin Y. Shuke N. Kaneko S. Okizaki A. Sato J. Aburano T. (2008). Cerebral control of bladder storage in patients with detrusor overactivity. Nucl. Med. Commun. 29, 1081–1085. 10.1097/MNM.0b013e328313bc13 18987529

[B71] Zhang H. Reitz A. Kollias S. Summers P. Curt A. Schurch B. (2005). An fMRI study of the role of suprapontine brain structures in the voluntary voiding control induced by pelvic floor contraction. Neuroimage 24, 174–180. 10.1016/j.neuroimage.2004.08.027 15588608

[B72] Zuo L. Chen J. Wang S. Zhou Y. Wang B. Gu H. (2019a). Intra- and inter-resting-state networks abnormalities in overactive bladder syndrome patients: An independent component analysis of resting-state fMRI. World J. Urol. 38, 1027–1034. 10.1007/s00345-019-02838-z 31172280

[B73] Zuo L. Zhou Y. Wang S. Wang B. Gu H. Chen J. (2019b). Abnormal brain functional connectivity strength in the overactive bladder syndrome: A resting-state fMRI study. Urology 131, 64–70. 10.1016/j.urology.2019.05.019 31150692

